# Computationally approximated solution for the equation for Henssge’s time of death estimation

**DOI:** 10.1186/s12911-019-0920-y

**Published:** 2019-10-28

**Authors:** Wolf Schweitzer, Michael J. Thali

**Affiliations:** 1 Department of Forensic Medicine and Imaging, Institute of Forensic Medicine, University of Zürich, Campus Irchel, Zürich, 8057 Switzerland; 2 Institute of Forensic Medicine, University of Zürich, Campus Irchel, Zürich, 8057 Switzerland

**Keywords:** Computational approximation, Estimation of early post-mortem interval, Forensic pathology, PHP

## Abstract

**Background:**

Time of death estimation in humans for the benefit of forensic medicine has been successfully approached by Henssge, who modelled body cooling based on measurements of Marshall and Hoare. Thereby, body and ambient temperatures are measured at the death scene to estimate a time of death based on a number of assumptions, such as initial body temperature and stable ambient temperature. While so far, practical use of the method resorted to paper print outs or copies of a nomogram using a ruler, increasingly, users are interested in computer or mobile device applications. We developed a computational solution that has been available online as a web accessible PHP program since 2005. From that, we have received numerous requests not so much to detail our code but to explain how to efficiently approximate the solution to the Henssge equation.

**Methods:**

To solve Henssge’s double exponential equation that models physical cooling of a body, it is sufficient to determine a difference term of the equation that will be close to zero for the correct time of death using a discrete set of all sensible possible solutions given that the modelled time frame has practical upper limits. Best post-mortem interval approximation yields minimal difference between equation terms

**Results:**

The solution is approximated by solving the equation term difference for a discrete set of all possible time of death intervals that are sensibly found, and by then determining the particular time of death where equation term difference is minimal.

**Conclusions:**

The advantage of a computational model over the nomogram is that the user is also able to model hypothermia and hyperthermia. While mathematically impossible to solve in a straightforward way, solutions to the Henssge equation can be approximated computationally.

## Background

The Henssge method for the estimation of time of death [1] a is popular tool for an early post-mortem period up to around two days.

It is predominantly used in forensic medicine, where a time of death estimate may have reconstructive and legal relevance [2, 3]. Thereby, body and ambient temperatures are measured at the death scene to estimate a time of death based on a number of assumptions, such as assuming an initial body temperature and assuming a stable ambient temperature. While the computational aspect can be addressed, it is important to point out that the method itself can only *estimate* a post-mortem interval, but it is not a *determination* or *calculation* of “the” post-mortem interval. Much rather, it is an *estimate* based on the input parameters that will be *approximated by calculation*.

The equation that defines this method was simplified by Henssge using the equation developed by Marshall and Hoare (1962) [4]. It mathematically models the temperature curves of actual measured physical cooling experiments. As that, it contains the relevant term that is to be determined by investigating authorities, the time of death estimate *t*_*i*_, twice, each time as part of an exponent.

To allow any user to practically obtain valid and fast solutions, nomograms were created by Henssge for easy replication through photocopies, and published [1]. These contain the relevant parameters in a form where the solution can be rapidly found by using a pencil and a ruler; however, one restriction that these nomograms contain is a fixed value for an assumed initial body temperature of 37,2^∘^*C*.

With the advent of increasingly more affordable computation power, calculating the solution of the Henssge equation seems to increasingly become an reality. Users of the conventional nomograms now increasingly require the same method to be made available on mobile devices.

Mathematically, there is no plain and easy solution for this double exponential equation. Since one cannot provide an analytical mathematical solution, approximating the result is the method of choice. We had programmed a fast approximation to practically correct, internally validated solutions using PHP (Hypertext Pre-Processor) in 2005 1. Internal validation was performed by cross-referencing arbitrary artificial numerical data across the paper nomograms and by performing step-wise equation term evaluation. After it had been extensively validated by our team then, it was externally validated in 2013 by a South African team [5].

Over the years, we have received numerous requests not so much to detail our code but to explain how to efficiently approximate the solution to the Henssge equation. This will allow users of various programming environments to efficiently program software for this there.

## Methods

The method chosen for this solution was that of approximation [6] rather than choosing more complex approaches [7] such as a step-wise iteration originally suggested [1].

For that, the problem’s ideal continuous solution space *t* is replaced with a finite discrete subset that contains discrete values across a practically sensible subspace *A* of all possible values of time, *t*_*A*_ [6, 8].

## Results

The equation to calculate the time of death estimate or interval *t*=*t*_*m*_−*t*_0_ for any given point in time *t*_*m*_ (time of examination, time of measurement of actual rectal temperature of the body) after time of death *t*_0_ bases on ambient temperature *T*_*U*_=*T*_*U*_(*t*_*m*_) that is implicitly assumed to be the same as the initial ambient temperature (so *T*_*U*_=*T*_*U*_(*t*_*m*_)=*T*_*U*_(*t*_0_) is set), an assumed initial body temperature at the time of death *T*_*D*_=*T*_*body*_(*t*_0_) (which is usually but not necessarily 37,2^∘^*C*), measured actual body core temperature *T*_*B*_=*T*_*body*_(*t*_*m*_) obtained from the environment adjacent to the body, outside of any present cover or clothing, an estimated or actual body weight *M* and an empirically determined corrective factor *f* that adapts the calculation to both body weight [9] and ambient conditions (basically modelling environmental aspects of cooling such as humidity and body covers) [10, 11].

For the correct solution for the time of death interval estimate *t* where factor *Z* is given in Eq. 3, the following term (up to an ambient temperature *T*_*U*_≤23.2^∘^*C*: Eq. 1; for any ambient temperature *T*_*U*_≥23.3^∘^*C*: Eq. 2) models the physical cooling according to Henssge [12, 13]:

1 $$ \frac{T_{B}-T_{U}}{T_{D}-T_{U}}=1.25 \cdot e^{Z \cdot {t}} - 0.25e^{5 \cdot Z \cdot t}   $$

2 $$ \frac{T_{B}-T_{U}}{T_{D}-T_{U}}=1.11 \cdot e^{Z \cdot {t}} - 0.11e^{10 \cdot Z \cdot t}   $$

3 $$ Z=(-1.2815 \cdot (f \cdot M^{-0.625})+0.0284)   $$

As the variable *t* cannot be isolated from these equations, they cannot be analytically solved for *t*.

The result can be approximated, however. Simply performing a forward calculation of all terms of that equation using a large vector containing a discretized set of *N* possible time of death estimates *t*_*A*_(*i*) with *i*={1,*N*} will yield *N* different equations.

The difference between both terms will contain the best approximation for *t*_*A*_(*i*) once that difference is minimal.

So, a vector *t*_*A*_ of discrete times of death *n*={1,*N*} is used to determine all *N* values for the difference vector *D* with Eq. 4 (*T*_*U*_≤23.2^∘^*C*) or Eq. 5 (*T*_*U*_≥23.3^∘^*C*):

4 $$\begin{array}{*{20}l} D=\frac{T_{B}-T_{U}}{T_{D}-T_{U}} &-\left(1.25e^{(-1.2815 \cdot (f \cdot M^{-0.625})+0.0284) \cdot t_{A}}\right. \\ &- \left. 0.25e^{5 \cdot (-1.2815 \cdot (f \cdot M^{-0.625})+0.0284) \cdot t_{A}}\right)  \end{array} $$

5 $$\begin{array}{*{20}l} D=\frac{T_{B}-T_{U}}{T_{D}-T_{U}} &-\left(1.11e^{(-1.2815 \cdot (f \cdot M^{-0.625})+0.0284) \cdot t_{A}} \right. \\ &- \left. 0.11e^{10 \cdot (-1.2815 \cdot (f \cdot M^{-0.625})+0.0284) \cdot t_{A}}\right)  \end{array} $$

For the presented best approximation, the one value *t*_*A*_(*i*) for which *D*(*i*)=*min*(|*D*|) is identified.

### Discretization and computational error

Practically, *N*≈300 discrete values are enough to cover the extent of the method between time of death estimates ranging from around an hour to approximately two days. The upper limit of around two days is a technical limitation in that body cooling then has mostly yielded such small differences between ambient and body temperature that estimation of time of death based on cooling or temperature becomes practically irrelevant.

A relatively small error will systematically result from the grain of the values: using decimal time values for *t*_*A*_, increasing *t*_*A*_(*i*) by tenths of an hour (6 min) may cause time of death estimate errors within maximally ∼1.5*%* of the usual 95% confidence interval the method reportedly gives (the smallest 95% confidence interval is 2,8 h [12, 14, 15]).

This computational error seems to be of no practical relevance. In the report the forensic pathologist will issue, the notation of results from time of death estimates are usually not given in decimal notation with a 1/10*th* h step. Issuing results with a maximal precision of a quarter of an hour, but normally with a maximal precision of 1/2 h, will even out the much smaller systematic result deviations incurred by discretization, rendering these effectively insignificant in relation to the application. Any systematic approximation derived error had neither been reported by users since the first installation of our web-based solution in 2005^2^, nor by the study validating our electronic solution in 2013 [5].

### Algorithm complexity

With regard to the discretization to a number of *N*≈300 discrete values, the algorithm will scale with *Θ*(*N*). Once defined, this algorithm conforms to a simple complexity of *Θ*(1). With that, it is laid out to require the same amount of time regardless of the value of the user’s input variables.

## Discussion

A particular advantage of a computational model over the nomogram is that the user is also able to model hypothermia and hyperthermia with practical ease. That is possible because the mathematical model of Henssge bases on Marshall and Hoare with a variable initial body temperature [4].

Otherwise, there is no practically relevant difference in terms of the results than when a correctly formatted [5] paper nomogram is used.

***Errors of the method and its application***

At this point, it may be relevant to consider that this paper only addresses the efficient computational approximation (and the error related to it) of the Henssge method for time of death estimation given its published technical aspects, as it is typically used in the early post-mortem period in forensic medicine.

Here, we do not address any potential errors related to the application of the Henssge method, These may, for example, be rooted in temperature measurement, body weight estimation. We also do not address any potential methodological errors inherent in the design or description of the method. These may, for example, stem from issuing a confidence interval based on two standard deviations as containing 95% (implied: 95,0 rather than ∼95,45*%*) [12, 14, 15], or from numerically simplifying the initially published 23,3^∘^*C*-cutoff for application of the correct equation [12, 15] to 23^∘^*C* (implied: 23,0) [16]. One study had examined the validity of the Henssge method and reported a violation of the predicted 95%-confidence intervals in 57,1% of their 84 cases. Also, they reported larger standard deviations from their own experimental data and a trend to overestimate the Henssge method based post-mortem interval in cases with high body mass and large body surface area [17].

Nevertheless, Henssge defined a scientifically based method for the estimation of post-mortem intervals, with a number of case specific variables to carefully consider, that is popular and accepted in the forensic medicine community. For the correct application of the method in any given case, original literature should be consulted [1–3, 12, 15, 16, 18, 19].

## Conclusions

While mathematically impossible to solve in a straightforward way, solutions to the Henssge equation can be approximated computationally by discretization with negligible error.

## Data Availability

Not applicable.

## Abbreviations

PHP: Hypertext pre-processor

## Footnotes

1 http://www.swisswuff.ch/calculators/todeszeit.php

2 http://www.swisswuff.ch/calculators/todeszeit.php
